# The Fungal Community Structure Regulates Elevational Variations in Soil Organic Carbon Fractions in a Wugong Mountain Meadow

**DOI:** 10.3390/jof10110772

**Published:** 2024-11-06

**Authors:** Jinping Wang, Jihong Yuan, Qiong Ren, Liyin Zhou, Huanhuan Zeng, Lujun Miao, Zhiyong Sun, Fang Wan, Yuanying Yan

**Affiliations:** 1 Jiangxi Key Laboratory for Intelligent Monitoring and Integrated Restoration of Watershed Ecosystem, Nanchang Institute of Technology, 289 Tianxiang Road, Nanchang 330099, China; wangjp0107@nit.edu.cn; 2 National Ecosystem Research Station of Jiangxi Wugong Mountain Meadow, Wetland Ecological Resources Research Center, Jiangxi Academy of Forestry, 1629 West Fenglin Street, Nanchang 330032, China; zhouly@jxlky.cn (L.Z.); zenghuanhuan1124@163.com (H.Z.); miaolujun@126.com (L.M.); andyd@163.com (Z.S.); zly800907@126.com (F.W.); yuanjh040@nenu.edu.cn (Y.Y.)

**Keywords:** SOC fractions, mountain meadow, elevation, fungal community, microbial residue carbon

## Abstract

Soil organic carbon (SOC) fractions are vital intrinsic indicators of SOC stability, and soil fungi are the key drivers of soil carbon cycling. However, variations in SOC fractions along an elevational gradient in mountain meadows and the role of the fungal community in regulating these variations are largely unknown, especially in subtropical areas. In this study, an elevation gradient experiment (with experimental sites at 1500, 1700, and 1900 m) was set up in a *Miscanthus sinensis* community in a meadow on Wugong Mountain, Southeast China, to clarify the effects of elevation on soil fungal community composition, microbial residue carbon, and SOC fractions. The results showed that the contribution of soil microbial residue carbon to SOC was only 16.1%, and the contribution of soil fungal residue carbon to SOC (15.3%) was far greater than that of bacterial residue carbon (0.3%). An increase in elevation changed the fungal community structure and diversity, especially in the topsoil (0–20 cm depth) compared with that in the subsoil (20–40 cm depth), but did not affect fungal residue carbon in the two soil layers. When separating SOC into the fractions mineral-associated organic carbon (MAOC) and particulate organic carbon (POC), we found that the contribution of MAOC (66.6%) to SOC was significantly higher than that of POC (20.6%). Although an increased elevation did not affect the SOC concentration, it significantly changed the SOC fractions in the topsoil and subsoil. The soil POC concentration and its contribution to SOC increased with an increasing elevation, whereas soil MAOC showed the opposite response. The elevational variations in SOC fractions and the POC/MAOC ratio were co-regulated by the fungal community structure and total nitrogen. Our results suggested that SOC stabilization in mountain meadows decreases with an increasing elevation and is driven by the fungal community structure, providing scientific guidance for SOC sequestration and stability in mountain meadows in subtropical areas.

## 1. Introduction

Since the beginning of the 20th century, global air temperature has risen due to the rise in atmospheric concentrations of CO_2_ and other greenhouse gases. The increase in global average temperature is over 1.1 °C now, and might reach 1.4–5.8 °C by the end of the 21st century [1], with the increases being greater in high-latitude and high-elevation regions. Disastrous consequences will occur without mitigating the global warming trend. Soil is the largest terrestrial organic carbon (C) reserve [2] and is central to climate change mitigation and ecosystem sustainability [3]. Globally, more activated C is stored in the soil than in the atmosphere and vegetation combined [4]. Therefore, small changes in soil organic carbon (SOC) have marked effects on atmospheric CO_2_ concentrations and, hence, on global climate [5]. Soil C decomposition caused by climate warming leads to a faster transfer of soil-stored C into the air, triggering a positive feedback loop of climate warming [6]. Enhancing soil C sequestration is a key strategy for mitigating the climate crisis [7]. The formation, decomposition, and transformation of soil C directly affect SOC sequestration [8], and SOC fractions directly affect the rate of soil C decomposition, thereby determining the stabilization of SOC [9,10]. Hence, research on the variation in SOC sources and SOC fractions is essential for understanding global C cycling and management of soil C sequestration.

The SOC can be separated into two physical fractions: particulate organic carbon (POC) and mineral-associated organic carbon (MAOC) [11]. POC is mainly formed from incompletely decomposed plant residues and is composed of lightweight fragments made up of large polymers [12], whereas MAOC is formed from single small molecules that originate from dead microbial biomass or leachate from plant residues and roots and is associated with silt- and clay-sized minerals [13,14]. Therefore, the stability of POC is low and it is easily decomposed by microorganisms, whereas MAOC has the opposite properties. Scientists have studied the effects of atmospheric CO_2_ concentration increases [15], climate warming [16], rainfall increases [17], and nitrogen deposition [18] on soil POC and MAOC and found that POC was more responsive to global change, whereas MAOC was generally less responsive [19]. However, these studies rarely involved variations in microbial communities even though microorganisms play a key role in soil C cycling. Microorganisms promote the formation and transformation of POC and MAOC, and microbial residues and secretions are important stable C sources for the soil C pool [20,21]. Microorganisms regulate the SOC quantity and quality, including the transformation of plant-derived C, the formation of microbial C, and the stabilization of microbial C [22]. There are two routes for transferring plant-derived C by microorganisms: first, microorganisms decompose and transform high-molecular-weight plant-derived substrates into low-molecular-weight compounds that can accumulate steadily in the soil by secreting extracellular enzymes; second, microorganisms synthesize their own biomass by assimilating plant-derived substrates; and ultimately, part of their necromass is stabilized in the soil. Hence, variations in microbial community structure, composition, and metabolic activities affect plant-derived C transformation and microbe-derived C formation [23,24], thus regulating SOC fractions and ultimately affecting soil C cycling and stabilization [25]. However, the effects of microbial community variations on microbial C generation and SOC fraction accumulation in changing environments remain unclear.

Microorganisms have key ecological functions in terrestrial ecosystems, and fungi play pivotal roles in organic matter decomposition and soil C cycling [26]. Compared with bacteria, fungi have a higher C/N ratio and some fungi species like white rot fungi tend to decompose complex substrates, such as lignin [27]. Among the soil fungi, saprotrophic fungi decompose soil organic substrates and release CO_2_ into the atmosphere, whereas mutualistic mycorrhizal fungi have the opposite effect, leading to enhanced C sequestration [28]. Numerous studies have indicated that the contribution of fungi to SOC accumulation is greater than that of bacteria [24,29,30,31], particularly in grassland ecosystems [32]. A meta-analysis conducted by Wang et al. [33] found that the accumulation efficiency of soil microbial residue C was higher in grasslands and farmlands than in forests; the contributions of microbial residue C to SOC were grassland (47–54%) > farmland (24–51%) > forests (34–44%), while fungal residue C contributed more to SOC (>65%) than bacterial residue C (32–36%). This latter observation could be attributed to the greater biomass of fungi and the lower decomposition rate of fungal cell compounds compared to those of bacteria [33]. Therefore, fungi play a key role in soil C storage and stabilization.

The ecological function, biomass production, and community distribution of fungi are readily affected by environmental changes [34,35] because environmental changes trigger variations in vegetation type and soil physicochemical properties, including soil water content, pH, C/N ratio, and temperature [36,37,38,39]. Variations in fungal community composition and ecological functions lead to changes in fungal residue C storage [40]. Different elevational gradients have significantly different climate, soil, and vegetation characteristics, thus creating complex environmental conditions, and are regarded as natural experiments for research on the ecological and evolutionary responses of microbial communities to changing environments [41,42,43]. The shift in the fungal community caused by changes in the environment along elevational gradients may lead to significant changes in litter decomposition and nutrient transformation [44,45], thus affecting the SOC fractions. Although the response of the fungal community composition and its biomass to elevation is clear, our knowledge of the relationship between the fungal community and fungal residue C at high elevations, which is the key to understanding how fungal communities affect SOC fractions, is limited.

Global grasslands cover approximately 40.5% of the Earth’s terrestrial area and store 34% of the terrestrial C stock [46]. Because of their large area and potential for C sequestration, grasslands are critical for the global C cycle [47]. Approximately 90% of the C in grasslands is stored belowground as root biomass and SOC, which are highly vulnerable to human disturbance and climate change [48]. Globally, grasslands have undergone severe degradation, and their biodiversity and ecosystem functions are declining, leading to reductions in SOC storage [49,50,51,52]. Much research has focused on the climatic effects on SOC variation, including warming, precipitation, drought, and nitrogen deposition, to slow the speed of soil C loss from grasslands [46,53,54]. However, such research has mainly been conducted in temperate regions because approximately 67% of global grasslands are distributed in temperate regions with arid, semi-arid, and cold climates [55]. The effect of elevation on SOC has been neglected in mountain meadows in subtropical humid regions, which is not helpful for an overall understanding of SOC variation in grasslands. Mountain meadows are special ecosystems with complex substrates that are dominant in soils and rely more on decomposition by fungi [56]. In addition, fungi are better adapted to the low-N and high-elevation environments of mountain meadows [57]. Recent research has found that increased fungal diversity promotes the stabilization efficiency of grass-litter-derived particulate organic matter (POM) but reduces that of mineral-associated organic matter (MAOM) [58], thus affecting SOC storage. Theoretically, variations in fungal metabolism and community composition caused by elevation inevitably affect the formation and stabilization of SOC; however, the processes at play are largely unknown.

The Wugong Mountain meadow is a typical subtropical mountain meadow distributed at an elevation range of 1500 to 1918 m. In this study, we investigated variations in the fungal community, microbial residue C, and SOC fractions in two soil layers (topsoil and subsoil) in response to elevational gradients in the Wugong Mountain meadow. We hypothesized that (i) elevation changes the fungal community diversity and structure, (ii) variation in fungal and bacterial residue C leads to a significant difference in SOC concentration along elevation gradients, (iii) the responses of the concentrations of the SOC fractions POC and MAOC to elevation are different along elevation gradients, and (iv) the difference in fungal community diversity or structure along elevation gradients alters the accumulation of SOC fractions. The goals of this study were to clarify the variations in SOC fractions along elevation gradients and reveal the fungal regulation of SOC fractions in subtropical mountain meadows. This study improves our understanding of the relationships between fungal communities and SOC fractions in humid mountain meadow ecosystems.

## 2. Materials and Methods

### 2.1. Study Area Description

Wugong Mountain is a national park in China located on the north side of the Luoxiao Mountains, reaching an elevation of 1918 m. It has a warm and humid monsoon climate zone. The climate in this region exhibits clear seasonal differences. The mean annual temperature is 15 °C, with the highest and lowest recorded temperatures of 39.7 °C and −9.3 °C. The average annual precipitation is approximately 1800 mm and the frost-free period is more than 200 days. The mountain meadow ecosystem is distributed on Wugong Mountain at an elevation of more than 1500 m, with a total area of 1569.24 hectares. The dominant herbaceous species are *Miscanthus sinensis* Andersson., *Miscanthus floridulus* (Labill.) Warburg ex K. Schumann., *Arundinella hirta* Steud., *Agrostis sozanensis* Hayata., *Pennisetum alopecuroides* (L.) Spreng., *Aster ageratoides* Turcz., *Osmunda japonica* hunb., *Spiraea japonica* L. f., *Senecio nemorensis* L., and *Anaphalis margaritacea* (L.) Benth. & Hook. f.

### 2.2. Experimental Design and Soil Sampling

We conducted our research in November 2022 at the Grassland Ecological Research Station of Wugong Mountain, Pingxiang, China. Samples were collected at three elevations (1500, 1700, and 1900 m) at approximately 200 m intervals on the sunny slope of Wugong Mountain. The dominant herbaceous *M. sinensis* community along an elevation gradient was selected as the sampling site. Three independent replicate sites (each 10 m × 10 m) were established at each elevation to account for the effects of elevation. The distance between replicate sites at the same elevation was greater than 50 m. Soil samples were collected from the topsoil (0–20 cm depth) and subsoil (20–40 cm depth) layers at each site. After litter removal, triplicate soil samples were collected from the same soil layer at each site and mixed to represent one pooled soil sample. Eighteen mixed soil samples (three elevations × three replicates × two soil layers) were collected in self-sealing bags for preservation. Each of the 18 soil samples was divided into two parts in the laboratory, one part being stored at −80 °C to measure fungal community diversity and structure, and one part being air-dried prior to measure SOC fractions and soil physicochemical properties.

### 2.3. Soil Physicochemical Analyses

The soil physicochemical properties were determined following the protocols of Ren et al. [59]. The soil pH was determined in a 1: 5 dilution of soil: water using a pH meter (PHS-3D, Shanghai Leica Instrument Co., Ltd., Shanghai, China). Soil total nitrogen (TN) was determined using an elemental analyzer (Vario MACRO cube; Elementar Trading Shanghai, Shanghai, China). Soil total phosphorus (TP) samples were digested with HClO_4_–H_2_SO_4_ and determined using molybdenum blue colorimetry. The OM and SOC concentrations were determined using potassium dichromate colorimetry.

### 2.4. SOC Fraction Analysis

Soil POC and MAOC were extracted using 5 g/L sodium hexametaphosphate according to the method described by Cambardella and Elliott [11]. Air-dried soil was sieved through a 2 mm sieve. A sub-sample (20 g) of sieved soil was placed in a plastic bottle. Then, 100 mL of sodium hexametaphosphate (5 g/L) was added, and a soil suspension was obtained after shaking for 18 h at 90 rpm. The soil suspension was passed through a 53 μm sieve and the residue repeatedly rinsed with distilled water until the water was clear. The materials that remained in the sieve and that passed through the sieve were collected and dried at 60 °C to a constant weight, representing POC and MAOC, respectively. Finally, the proportions of their contents relative to the soil dry weight were calculated.

### 2.5. Soil Microbial Residue C Analyses

Muramic acid (MurN) is uniquely derived from bacterial peptidoglycans and glucosamine (GluN) predominantly originates from chitin in fungal cell walls. Hence, the bacterial and fungal residue C concentrations were calculated from the MurN and GluN concentrations, respectively. The concentrations of individual amino sugars (MurN, GluN, and galactosamine (GalN)) were determined according to the method described by Indorf et al. [60]. A sub-sample (0.2 g) of air-dried soil was placed in a hydrolytic tube, and 5 mL of HCl (6 mol/L) was added. The hydrolytic tube was incubated in an oven at 105 °C for 48 h to fully hydrolyze the soil sub-sample. Chromatographic separations were performed on a HyperClone C_18_ column (150 mm length × 4.6 mm diameter) at 35 °C, using an Agilent 6890 gas chromatograph with a flame ionization detector set at 445 nm emission and 330 nm excitation wavelengths. The concentrations of individual amino sugars (MurN, GluN, and GalN) were quantified using the internal standard, myo-inositol.

### 2.6. Soil DNA Extraction, PCR Amplification, and Illumina Sequencing

Soil fungal DNA was extracted from three independent 0.50 g sub-samples of soil, which had been stored at −80 °C, using a metagenomic DNA Extraction Kit (GENErary), according to the protocol of the manufacturer. The quality and concentration of the extracted DNA were assessed using 1% agarose gel electrophoresis and UV spectrophotometry (AA900T; Perkin Elmer, Norwalk, CA, USA), respectively. The DNA extracts were diluted and stored at −20 °C. The fungal rRNA internal transcribed spacer ITS-1 region was amplified using the primers ITS1F (5ʹ-CTTGGTCATTTAGAGGAAGTAA-3ʹ) and ITS2 (5ʹ-GCTGCGTTCTTCATCGATGC-3ʹ) [61]. PCR amplification was performed as described by Ren et al. [59]. The amplicons were sequenced using an Illumina MiSeq platform (EDC-810; Beijing Bai Mai Biotechnology Co., Ltd., Beijing, China).

Raw sequence data (FASTQ files) were quality-filtered using the Trimmomatic tool and merged using FLASH v1.2.11 software according to three criteria [62]. Sequences with ambiguous bases were excluded from the analysis. Trimmed and unique sequences were clustered using USEARCH at a 97% similarity level to generate operational taxonomic units (OTUs). The taxonomic identity of each phylotype was assigned using a QIIME2 comparison against the UNITE database (https://unite.ut.ee/ (accessed on 18 July 2023)). The diversity and richness indices (Chao1, Simpson, Shannon, and Pielou) of the fungal communities were calculated using QIIME2.

### 2.7. Statistical Analysis

Duncan’s multiple range test (*p* < 0.05) was performed on SOC fractions, microbial residue C, fungal α-diversity indices, the relative abundances of fungal species, and soil physicochemical properties at different elevations in topsoil or subsoil. Multivariate two-way (interaction) analysis of variance (MANOVA) was used to analyze the effects of elevation and soil depth on SOC fractions, microbial residual C, and the microbial residual C: SOC ratio. The results were expressed as the mean ± standard error (*n* = 3). Fungal OTU numbers were normalized using log (OTUs + 1), and redundancy analysis (RDA) of the fungal community and soil chemical factors was conducted using a Monte Carlo test based on an axis length of <4. The statistical significance test included 999 permutations. Non-metric multidimensional scaling (NMDS) was used to illustrate the clustering of different samples and further reflect the fungal community structure based on Bray–Curtis distances at the OTU level.

The relationship between SOC fractions and functional groups of fungi, fungal community composition, and fungal α-diversity indices were investigated using the “pheatmap” package in R, while the correlation coefficients between SOC fractions and soil physicochemical properties were determined using the “corrplot” package in R. Structural equation modeling (SEM) was used to estimate the influence pathway of elevation on SOC fractions and the POC/MAOC ratio. All analyses were conducted using R (v3.6.2).

## 3. Results

### 3.1. Variation in Soil Physicochemical Properties Along an Elevation Gradient

Elevation had no significant effect on soil pH or OM concentration but significantly affected soil TN concentration, TP concentration, and the C/N ratio, especially in the topsoil (0–20 cm) compared with the subsoil (20–40 cm) (Table 1). Soil TN and TP concentrations decreased with an increasing elevation, and the decreases were significant at elevations of 1700 and 1900 m (*p* < 0.05) compared with that at 1500 m, while the soil C/N ratio increased with an increasing elevation. The SOC, TN, and TP concentrations in the topsoil were significantly higher than those in the subsoil.

### 3.2. Variation in SOC Fractions Along an Elevation Gradient

**Figure 1:**
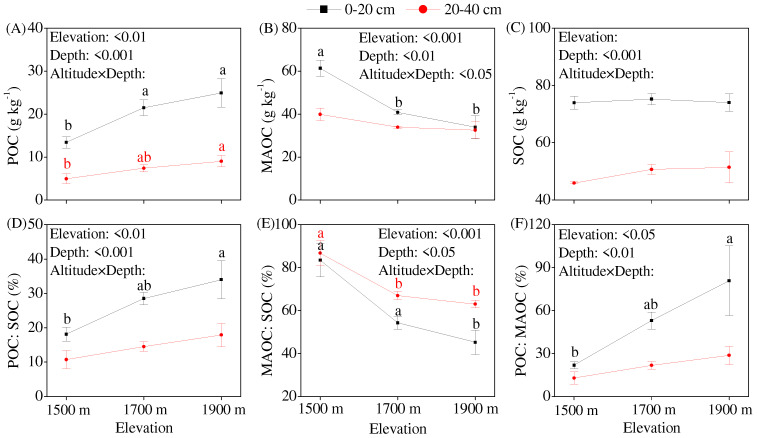
Variation in soil organic carbon (SOC) fractions along an elevation gradient in a mountain meadow. (**A**) POC concentration, (**B**) MAOC concentration, (**C**) SOC concentration, (**D**) POC: SOC ratio, (**E**) MAOC: SOC ratio, (**F**) POC: MAOC ratio. Note: POC, particulate organic carbon; MAOC, mineral-associated organic carbon; the same lowercase letter indicates no significant differences (*p* < 0.05) among elevation gradients within the same soil layer.

Although an increasing elevation did not significantly affect the SOC concentration, it did significantly affect the SOC fractions (Figure 1A–C). As for the SOC fraction, the POC concentration increased, and the MAOC concentration decreased in response to an increasing elevation. This trend was more evident in the topsoil (0–20 cm) than in the subsoil (20–40 cm) (Figure 1A,B). In the topsoil, the soil POC concentration increased significantly, whereas the soil MAOC concentration decreased significantly at an elevation of 1700 m compared with that at 1500 m. The increase in the contribution of POC to SOC (POC/SOC ratio) and the decrease in the contribution of MAOC to SOC (MAOC/SOC ratio) reached significant levels at an elevation of 1900 m compared to that at 1500 m (Figure 1D,E). However, in the subsoil, the increase in POC concentration and decrease in the MAOC/SOC ratio reached significant levels when the elevation reached 1700 m and 1900 m, respectively. The soil layer significantly affected the SOC and its fractions, indicating that the concentrations of SOC and its fractions were higher in the topsoil than in the subsoil. The average contribution of MAOC to SOC was 66.6% (ranging from 34.3% to 97.4%), whereas that of POC to SOC was 20.6% (ranging from 7.6% to 44.3%). The POC/MAOC ratio increased with an increasing elevation, and the increased value reached a significant level at an elevation of 1900 m compared with that at 1500 m in the topsoil (Figure 1F).

### 3.3. Variation in Soil Microbial Residue C Along an Elevation Gradient

**Figure 2:**
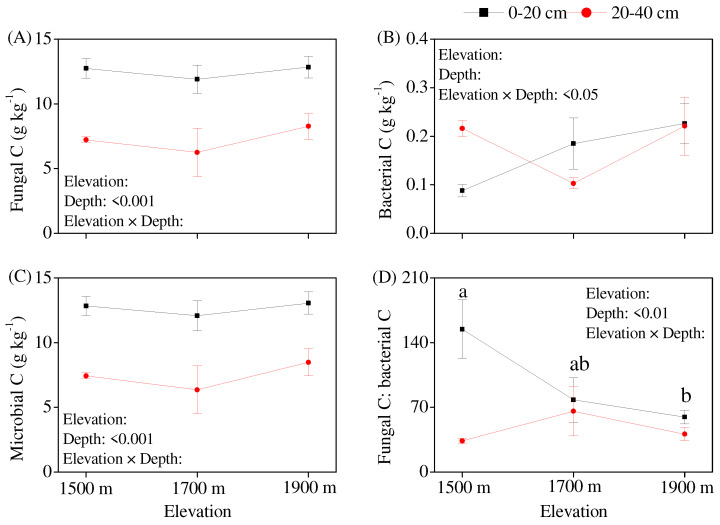
Variation in soil microbial residue C along an elevation gradient in a mountain meadow. (**A**) Fungal residue C concentration, (**B**) Bacterial residue C concentration, (**C**) Microbial residue C concentration, (**D**) Fungal residue C: Bacterial residue C ratio. Note: The same lowercase letter indicates no significant differences (*p* < 0.05) among elevation gradients within the same soil layer.

**Figure 3:**
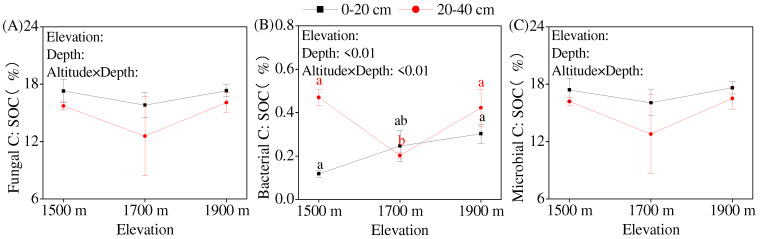
The contribution of soil microbial residue C to soil organic carbon (SOC). (**A**) Fungal residue C: SOC ratio, (**B**) Bacterial residue C: SOC ratio, (**C**) Microbial residue C: SOC ratio. Note: The same lowercase letter indicates no significant differences (*p* < 0.05) among elevation gradients within the same soil layer.

The effects of elevation on soil microbial, fungal, and bacterial residue C were not significant (Figure 2A–C). The fungal/bacterial residue C ratio decreased in the topsoil in response to an increasing elevation, reaching a significant level at an elevation of 1900 m compared to that at 1500 m (Figure 2D). The soil layer had significant effects on microbial and fungal residue C but had no significant effect on bacterial residue C, with the concentrations of microbial and fungal residue C being significantly higher in the topsoil than in the subsoil. The contributions of microbial residue C to SOC were not significantly affected by elevation (Figure 3A–C). The contributions averaged 16.1% (ranging from 6.8% to 20.7%) (Figure 3C), and 98.1% of microbial residue C was contributed by fungal residue C. The contribution of fungal residue C to SOC (15.8%) was far higher than that of bacterial residue C (0.3%) (Figure 3A,B).

### 3.4. Variation in Soil Fungal Community α-Diversity Along an Elevation Gradient

Soil fungal community diversity was significantly affected by elevation, especially in the topsoil (Table 2). In the topsoil, the fungal community Chao1 index increased with an increasing elevation, reaching a significant level at an elevation of 1700 m compared with that at 1500 m. The Simpson and Pielou indices of the fungal community decreased significantly at an elevation of 1900 m, while the Shannon index increased significantly at an elevation of 1700 m compared with that at 1500 m. In the subsoil, only the Chao1 index showed significant differences among the different elevations, and the response of the Chao1 index to an increasing elevation in the subsoil was similar to that of the topsoil; however, the increased value of the Chao1 index reached significance only when the elevation reached 1900 m when compared with that at 1500 m.

### 3.5. Variations in Soil Fungal Community Composition and Structure Along an Elevation Gradient

**Figure 4:**
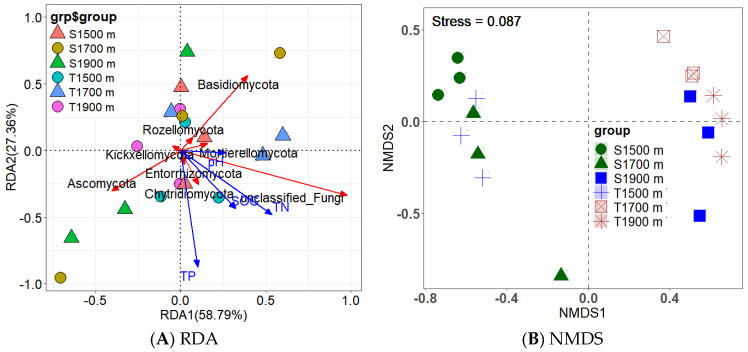
Redundancy analysis (RDA) and non-metric multidimensional scaling (NDMS) analysis of soil fungal community structure at different elevations. (**A**) RDA analysis, (**B**) NMDS analysis. Note: SOC, soil organic carbon; TN, total nitrogen; TP, total phosphorus.

Analysis of the seven main fungi identified at the phylum level revealed that the relative abundances of *Ascomycota* and *Mortierellomycota* in the topsoil were significantly different among the various elevations; the value of *Ascomycota* was the lowest, and that of *Mortierellomycota* was the highest at an elevation of 1700 m (Table 3). In the subsoil, the abundance of *Kickxellomycota* was affected by elevation, with the relative abundance of *Kickxellomycota* increasing with an increasing elevation and the increased value reaching a significant level at an elevation of 1900 m compared with that at 1500 m. *Glomeromycota* was not detected in soil. RDA showed that 87.15% of the variation in fungal community composition could be explained by soil chemical properties, with the fungal community composition mainly affected by soil TN and SOC (Figure 4A). Among the fungal phyla, the abundances of *Chytridiomycota* and unclassified_Fungi were positively correlated with soil TN and SOC (*p* < 0.05), whereas that of *Kickxellomycota* was negatively correlated with soil TN and SOC (*p* < 0.05).

The stress value of the NMDS analysis based on the Bray–Curtis distance was 0.087. The fact that this value was <0.200 indicates the reliability of the analysis. For the fungal community structure of the topsoil, the soil samples collected at an elevation of 1700 m were close to those collected at 1900 m but relatively far from those collected at 1500 m. Meanwhile, in the subsoil, the distances between the soil samples collected at an elevation of 1700 m were close to those collected at 1500 m but relatively far from those collected at 1900 m. Hence, elevation had important effects on soil fungal community structure, with the effects being greater in the topsoil than in the subsoil (Figure 4B).

### 3.6. Factor Analysis of SOC Fractions

**Figure 5:**
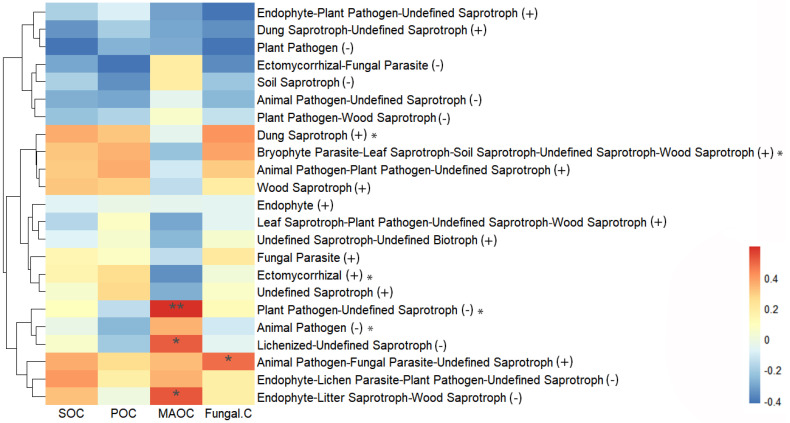
Correlation analysis between soil organic carbon (SOC) fractions and functional groups of fungi. Note: POC, particulate organic carbon; MAOC, mineral-associated organic carbon; symbol between parentheses describes the effect of elevation on fungal functional groups, “−” denotes a negative relationship between fungal functional group and increasing elevation, “+” means a positive relationship, and “*” outside parentheses means the elevation effect was significant; ”*” and “**” in the heat map indicate the correlation are significant at 0.05 and 0.01.

**Figure 6:**
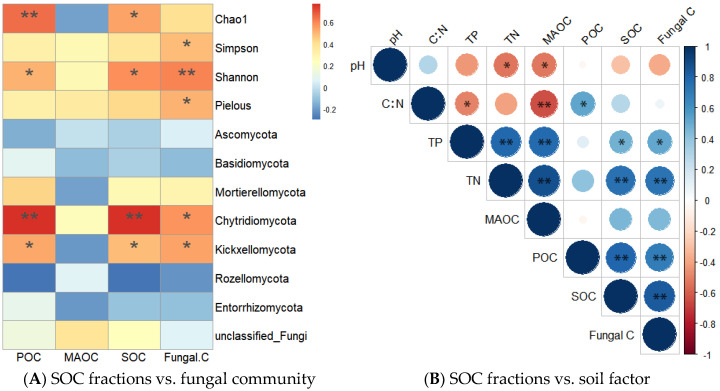
Correlation analysis between soil organic carbon (SOC) fractions and fungal community or soil factor. Note: POC, particulate organic carbon; MAOC, mineral-associated organic carbon; TN, total nitrogen; TP, total phosphorus; *, *p* < 0.05; **, *p* < 0.01.

Heat map analysis showed that soil MAOC was positively correlated with the relative abundance of saprophytic fungi, such as plant pathogen–undefined saprotrophs (*r* = 0.599, *p* < 0.01), endophyte–litter saprotroph–wood saprotrophs (*r* = 0.521, *p* < 0.05), and lichenized–undefined saprotrophs (r = 0.511, *p* < 0.05) (Figure 5). The relative abundances of these saprophytic fungi were negatively affected by an increase in elevation. Correlation analysis showed that soil POC and SOC were positively correlated with the fungal Chao1 index (*r* = 0.649, *p* < 0.01; *r* = 0.540, *p* < 0.05, respectively), Shannon index (*r* = 0.510, *p* < 0.05; *r* = 0.579, *p* < 0.05, respectively), the abundance of the fungal phylum *Chytridiomycota* (*r* = 0.769, *p* < 0.01; *r* = 0.769, *p* < 0.01, respectively), and the abundance of *Kickxellomycota* (*r* = 0.533, *p* < 0.05; *r* = 0.483, *p* < 0.05, respectively). Furthermore, fungal residue C was positively correlated with the fungal Shannon index (*r* = 0.606, *p* < 0.01), Simpson index (*r* = 0.573, *p* < 0.05), Pielou index (*r* = 0.536, *p* < 0.05), *Chytridiomycota* abundance (*r* = 0.505, *p* < 0.05), and *Kickxellomycota* abundance (*r* = 0.505, *p* < 0.05) (Figure 6A).

**Figure 7:**
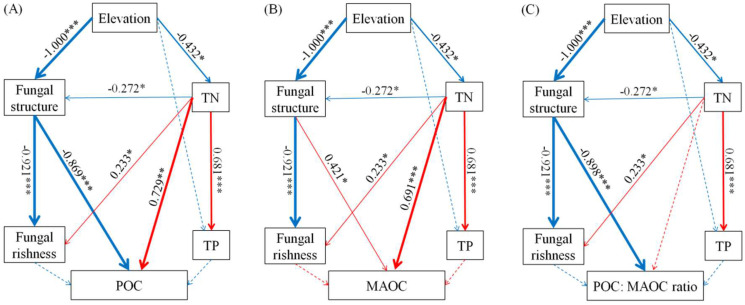
Structural equation model (SEM) of soil organic carbon fractions. Note: POC, particulate organic carbon; MAOC, mineral-associated organic carbon; TN, total nitrogen; TP, total phosphorus; *, *p* < 0.05; **, *p* < 0.01; ***, *p* < 0.001; (**A**) X^2^ = 5.429, CMIN/DF = 1.357, *p* = 0.246, GFI = 0.920, CFI = 0.985, RMSEA = 0.145; (**B**) X^2^ = 5.688, CMIN/DF = 1.422, *p* = 0.224, GFI = 0.917, CFI = 0.984, RMSEA = 0.158; (**C**) X^2^ = 5.729, CMIN/DF = 1.2432, *p* = 0.220, GFI = 0.916, CFI = 0.980, RMSEA = 0.159.

Correlation analysis between SOC fractions and soil physicochemical properties indicated that MAOC was positively correlated with TP (*r* = 0.780, *p* < 0.01) and TN (*r* = 0.874, *p* < 0.01) but negatively correlated with the pH value (*r* = −0.524, *p* < 0.05) and C/N ratio (*r* = −0.641, *p* < 0.01). Soil POC was positively correlated with the C/N ratio (*r* = 0.525, *p* < 0.05), whereas SOC was positively correlated with TP (*r* = 0.477, *p* < 0.05) and TN (*r* = 0.742, *p* < 0.01) (Figure 6B). Structural equation modeling (SEM) showed that the elevational variation in the POC: MAOC ratio was regulated by soil fungal community structure, while the elevational variations in POC and MAOC were co-regulated by soil fungal community structure and TN (Figure 7A–C).

## 4. Discussion

### 4.1. Contribution of Fungi to the Soil C Pool Was Far Higher than That of Bacteria

The soil C pool is mainly derived from plant and microbial residue C, and most studies have found that the contribution of soil microbial residue C to SOC is equivalent to or even greater than that of plant residue C [32,63,64]. However, the published results for soil MAOC are open to debate, as stable isotopes reveal that fungal residues contribute more to mineral-associated organic matter (MAOM) pools than plant residues [63]. The stoichiometric approach showed that the contribution of plant residues to MAOM (53–66%) exceeds that of microbial residues (34–47%) [65]. The present study showed that the contribution of microbial residue C to SOC was only 16.1%, indicating that more C was derived from plant-derived C inputs (Figure 3C), which is consistent with the results of previous studies [65,66]. Generally, plant residues are less well assimilated by microorganisms in harsh ecosystems (e.g., under cold or hypoxic conditions), leading to increased plant-derived C storage in the soil [67]. In the mountain meadow, the low temperature reduced microbial activity, leading to a lower assimilation of plant residues. Most studies have shown that fungal residue C contributes more to the soil C pool than bacterial residue C [32]. Our study showed that the contribution of fungal residue C to SOC was much higher than that of bacterial residue C (Figure 3A,B). This discrepancy might be attributed to the lower decomposition rate of cellular compounds and the higher C-use efficiency of fungi than that of bacteria [21]. Moreover, fungi readily decompose grass litter with a high lignin content, which is difficult to decompose, and hence are better adapted to low-nutrient environments [4,27], leading to far higher fungal biomass than bacterial biomass, resulting in far higher fungal residue C than bacterial residue C in a mountain meadow. However, a study conducted in an alpine meadow (elevation of 4750 m) on the Tibetan Plateau showed that the contributions of fungal and bacterial residues to the soil C pool (5.7% and 7.5%, respectively) were not very different [68]. A possible explanation is that the harsh climatic environment created by a very high elevation inhibits plant growth and greatly reduces the C input from plants to the soil system [69]; however, most fungi cannot survive such harsh environments.

### 4.2. Elevation Changed the Soil Fungal Community Structure and Diversity

Microorganisms are important in regulating biogeochemical cycling and maintaining ecosystem functions [70]. Fungi maintain various ecosystem processes, including belowground C transportation, plant litter decomposition, and plant growth [71,72,73]. Compared with bacteria, fungi can decompose recalcitrant organic materials better [26] and adapt better to soil conditions of low nitrogen and high C:N ratios [57]. Fungi are sensitive to environmental changes [74], and variations in the soil fungal community can affect soil C storage [63]. Published reports on the effects of elevation on soil fungal communities are inconsistent and may be related to different ecosystem types. In forest ecosystems, elevation has little effect on soil fungal community composition [75] or affects soil fungal community structure but not diversity [76]. Elevation affects soil fungal communities in a meadow ecosystem [77], which is consistent with the results of our study. The variations in fungal community diversity, composition, and structure with an increasing elevation verified hypothesis (i) (Table 2 and Table 3, Figure 4B). The effect of elevation on the soil fungal community structure is driven by vegetation type, soil properties, and climate change [76,77]. Among the soil properties, SOC, TP, TN, the C/N ratio, pH, temperature, humidity, and bulk density significantly affect soil fungal community composition [75,76,77,78]. In the present study, elevation-based changes in soil fungal community structure were driven by soil TN and SOC (Figure 4A). Nitrogen is the most important element in microbial cells; therefore, the lower soil TN concentration at higher elevations increases the C/N value, leading to greater constraints on microbial growth and reproduction by soil nitrogen (N), ultimately changing the soil fungal community. We also found that soil fungal community diversity was significantly and positively correlated with soil POC (Figure 6A). POC is composed of plant residues that are easily decomposed, with higher soil POC concentrations at higher elevations indicating that more C is available for fungi, which can relieve competitive pressure on fungi and lead to an increase in fungal community diversity. In addition, we found that the fungal community diversity in the topsoil was higher than that in the subsoil (Table 2), a finding which was consistent with that of Yang et al. [79], who found that, except for nutrient factors, the subsoil created an enclosed microhabitat, which blocked the immigration of exogenous fungal species, maintaining an ecologically specialized and less diverse local community. Topsoil is more susceptible to external environmental changes, such as plant litter input and temperature changes; therefore, the effect of elevation on fungal community structure and diversity was higher in topsoil than in subsoil in the present study.

### 4.3. Elevation Did Not Change Soil Fungal Residue C Concentration

Generally, harsher environments created at higher elevations will regulate the fungal community, decrease fungal C-use efficiency, and reduce plant residue decomposition by fungi, ultimately leading to a decrease in fungal biomass and fungal residue C. Interestingly, no variation in fungal residue C concentrations was found in response to an elevation gradient (Figure 3A). However, the fungal community changed in our study, a finding which was inconsistent with hypothesis (ii). Accumulation of fungal residue C in soil is caused by the formation of fungal residues and by the decomposition of fungal residues [21]. Soil temperature decreased with an increasing elevation, which reduced the formation of fungal residue; however, the decomposition of fungal residue was also reduced. Therefore, the accumulation efficiency of fungal residue increased [56]; ultimately, the soil fungal residue C concentration did not change along the elevation gradients. Furthermore, we found that soil fungal residue C was significantly and positively correlated with fungal community diversity and some saprophytic fungi such as *Kickxellomycota* (Figure 5 and Figure 6A). Hence, we hypothesized that certain fungal phyla, such as *Kickxellomycota*, adapted well to the environment at higher elevations and compensated for the decrease in fungal residues caused by the biomass reduction in other fungi. *Zoosporic* fungi phylum *Chytridiomycota* was not affected by elevation, but it was significantly and positively correlated with soil fungal residue C, indicating that *Chytridiomycota* might be one of the main fungal phyla that produce fungal residue C at higher elevations. *Glomeromycota* is an important fungal phylum that contributes to soil carbon sequestration in grassland. However, it was not detected, which was attributed to the primers used in our study. Fungal community richness (Chao1 index) and the relative abundance of the phyla *Kickxellomycota* and *Chytridiomycota* increased, whereas the Simpson index decreased, and the fungal community structure changed significantly at higher elevations (Table 2 and Table 3, Figure 4B). Soil properties are considered important factors affecting the accumulation of fungal residue C. A meta-analysis showed that the soil C/N ratio controls the accumulation of soil microbial residue C [80]. However, no significant correlation was observed between soil microbial residue C and the C/N ratio in our study (Figure 6B). Recently, a comprehensive global analysis found that soil N is one of the most important factors affecting the accumulation of microbial residual C [81]. Our study results also showed that the accumulation of fungal residue C was significantly correlated with soil N concentration (Figure 6B).

### 4.4. Elevation Did Not Change SOC Concentration but Significantly Affected SOC Fractions

The relative concentrations of the SOC fractions are different among terrestrial ecosystems, which reflects SOC stabilization. More SOC is stored as POM in forest ecosystems, whereas more SOC is stored as MAOC in grassland ecosystems [27]. Our study showed that 66.6% of SOC was stored as MAOC in the mountain meadows (Figure 1D), confirming the findings of Cotrufo et al. [27]. The effects of elevation on the SOC concentrations were variable. Garten and Hanson [82] found that SOC storage increased with an increasing elevation, while Djukic et al. [83] reported that SOC storage increased with an increasing elevation below 1500 m but decreased with an increasing elevation above 1500 m. The elevation of the mountain meadow in our study ranged from 1500 m to 1900 m, and our data showed that SOC and microbial residue C concentrations did not increase or decrease with an increasing elevation (Table 1, Figure 3C). The effect of elevation on soil C concentration may be related to vegetation type; the soil C concentration increased with an increasing elevation in bamboo forests, but this trend was not observed in grasslands [84]. Although elevation did not change the SOC concentration in the present study, it significantly affected SOC fractions, increasing the POC concentration and POC/MAOC ratio, while decreasing the MAOC concentration with an increasing elevation (Figure 1A, B, and F). This indicated that SOC stabilization decreased with an increasing elevation in subtropical mountain meadows, thus verifying hypothesis (iii).

The SOC fraction POC mostly comprises undecomposed or semi-decomposed macromolecular plant structural materials such as lignin and semi-lignin [12,85]. Lignin turnover is mainly controlled by temperature and requires moderate-to-high temperatures [86]. The lower soil temperature at higher elevations decreased lignin decomposition by fungi, leading to a greater accumulation of macromolecular plant structural materials; thus, the soil POC concentration increased with an increasing elevation. The SOC fraction, MAOC, consists of low-molecular-weight compounds derived from microorganisms and plants, such as microbial residues, plant lipids, and polysaccharides [13,14]. Decreases in soil temperature, weathering, and N supply at higher elevations slow the rates of microbial decomposition and C-use efficiency, decreasing the decomposition of macromolecular plant residues. Therefore, more macromolecular plant residues were stored in the soil, and fewer low-molecular-weight plant residues were formed and stored in the soil at high elevations, ultimately contributing to increased soil POC concentrations and decreased soil MAOC concentrations at higher elevations. Moreover, higher POC concentrations at higher elevations might be attributed to the increase in fungal community diversity, because greater diversity can promote the stabilization efficiency of grass-litter-derived POM [58]. In our study, SEM analysis verified that elevation affected SOC fractions and the POC/MAOC ratio by changing the fungal community structure and decreasing the soil TN content (Figure 7A–C), thus verifying hypothesis (iv).

Generally, the soil C/N ratio is inversely proportional to the decomposition rate of organic matter by microorganisms. The increase in the soil C/N ratio with an increasing elevation in our study (Table 1) indicates that the decomposition rate of organic matter by microorganisms was lower at higher elevations. This might be attributed to the decrease in the abundance of certain saprophytic fungi, such as endophyte–litter saprotroph–wood saprotrophs, because the abundance of this fungus was negatively affected by an increasing elevation (Figure 5). Lower N contents and soil temperatures at higher elevations limit the survival of saprophytic fungi. A significant and positive correlation between MAOC concentration and endophyte–litter saprotroph–wood saprotroph abundance suggested that the decrease in soil MAOC concentration at higher elevations was related to the decreased amount of endophyte–litter saprotroph–wood saprotrophs (Figure 5). Moreover, the accumulation of MAOC in the soil is related to soil mineral preservation, the rate of which increases with an increasing degree of weathering [87]. Lower concentrations of soil nutrients P and N (Table 1) indicated lower rates of soil weathering and organic matter decomposition at higher elevations, respectively, and lower soil weathering rates resulted in fewer active minerals (clay and metal oxides), which is not conducive to the formation and storage of MAOC. Furthermore, our results showed that the soil C/N ratio was significantly and positively correlated with POC but negatively correlated with MAOC (Figure 6B). This is not surprising because the main POC materials such as lignin have high C/N ratios (10–40), whereas the organic matter of MAOC has low C/N ratios [12,88,89].

## 5. Conclusions

Although considerable literature exists on the response of grassland SOC to environmental changes, the effects of elevation on SOC fractions and microbe-driven mechanisms in subtropical mountain meadows are relatively unclear. We designed the experiments described in the current study to address this shortcoming and obtained interesting results. We found that the contribution of microbial residue C to SOC and MAOC was less than 20%, largely due to fungal residue C, and not bacterial residue C, suggesting that the decomposition and transformation of SOC in subtropical mountain meadows were largely dominated by fungi rather than bacteria. Generally, variations in microbial communities affect the accumulation of microbial residues. However, in our study, an increased elevation significantly affected the fungal community composition, while it did not affect the accumulation of fungal residue C, suggesting that the change in the microbial community did not necessarily affect the accumulation of microbial residues. Furthermore, we found that an increased elevation significantly affected SOC fractions but did not affect the SOC concentration. The variation in the fungal community structure and TN with an increasing elevation maintained the accumulation of the fungal residue but altered the SOC fractions and stabilization.

## Figures and Tables

**Table 1:** Soil physicochemical properties along an elevation gradient in a mountain meadow.

Soil Depth	Elevation	pH	OM (g/kg)	TN (g/kg)	TP (g/kg)	C:N
0–20 cm	1500 m	5.31	127.44	4.80 a	0.82 a	15.62 b
	1700 m	5.46	129.61	3.68 b	0.53 b	20.43 a
	1900 m	5.43	127.59	3.40 b	0.55 b	21.73 a
20–40 cm	1500 m	5.49	79.18	2.88	0.59 a	16.00 b
	1700 m	5.48	87.36	2.62	0.35 b	19.34 a
	1900 m	5.49	88.63	2.54	0.44 ab	20.18 a

Note: SOC, soil organic carbon; TN, total nitrogen; TP, total phosphorus; the same lowercase letter indicates no significant differences (*p* < 0.05) among elevation gradients within the same soil layer.

**Table 2:** Soil fungal community α-diversity along an elevation gradient in a mountain meadow.

Indices	0–20 cm			20–40 cm			Significance		
	1500 m	1700 m	1900 m	1500 m	1700 m	1900 m	Elevation	Depth	Elevation × Depth
Chao1	292 b	378 a	430 a	213 b	275 b	386 a	0.000	0.001	0.420
Simpson	0.979 a	0.981 a	0.965 b	0.948	0.892	0.929	0.729	0.084	0.639
Shannon	6.445 b	6.908 a	6.421 b	5.712	5.149	5.682	0.993	0.011	0.432
Pielou	1.136 a	1.166 a	1.059 b	1.065	0.917	0.956	0.497	0.045	0.492

Note: The same lowercase letter indicates no significant differences (*p* < 0.05) among elevation gradients within the same soil layer.

**Table 3:** Variation in soil fungal phyla along an elevation gradient in a mountain meadow.

Phylum	0–20 cm			20–40 cm			Significance		
	1500 m	1700 m	1900 m	1500 m	1700 m	1900 m	Elevation	Depth	Elevation × Depth
*Ascomycota*	72.98 a	64.69 b	73.02 a	70.90	71.96	77.33	0.45	0.47	0.67
*Basidiomycota*	19.18	24.13	20.41	21.82	21.24	17.39	0.71	0.77	0.78
*Mortierellomycota*	1.90 b	3.24 a	2.41 b	2.10	1.97	2.44	0.20	0.21	0.08
*Chytridiomycota*	0.17	0.32	0.16	0.03	0.02	0.06	0.12	0.00	0.03
*Kickxellomycota*	0.09	0.16	0.21	0.04 b	0.10 ab	0.12 a	0.02	0.03	0.81
*Rozellomycota*	0.00	0.00	0.00	0.09	0.00	0.00	0.40	0.34	0.40
*Entorrhizomycota*	0.01	0.01	0.01	0.02	0.00	0.00	0.62	0.95	0.41
unclassified_Fungi	5.66	7.46	3.77	5.00	4.71	2.66	0.13	0.19	0.72

Note: The same lowercase letter indicates no significant differences (*p* < 0.05) among elevation gradients within the same soil layer.

## Data Availability

The original contributions presented in this study are included in the article, and further inquiries can be directed to the corresponding authors.
